# Recent Progress in Flexible Surface Acoustic Wave Sensing Technologies

**DOI:** 10.3390/mi15030357

**Published:** 2024-02-29

**Authors:** Chenlong Liang, Cancan Yan, Shoupei Zhai, Yuhang Wang, Anyu Hu, Wen Wang, Yong Pan

**Affiliations:** 1 Institute of Acoustics, Chinese Academy of Sciences, Beijing 100190, China; liangchenlong22@mails.ucas.ac.cn (C.L.); zhaishoupei@mail.ioa.ac.cn (S.Z.); huanyu@mail.ioa.ac.cn (A.H.); 2 The School of Electronic, Electrical and Communication Engineering, University of Chinese Academy of Sciences, Beijing 100049, China; 3 State Key Laboratory of NBC Protection for Civil, Beijing 102205, China; ccy805905145@163.com; 4 School of Chemistry and Chemical Engineering, Shanxi University, Taiyuan 030006, China; 202222902021@email.sxu.edu.cn

**Keywords:** flexible sensing technology, substrate material, piezoelectric thin films, physical flexible SAW sensors, structural design of interdigital transducers

## Abstract

In this work, the major methods for implementing flexible sensing technology—flexible surface acoustic wave (SAW) sensors—are summarized; the working principles and device characteristics of the flexible SAW sensors are introduced; and the latest achievements of the flexible SAW sensors in the selection of the substrate materials, the development of the piezoelectric thin films, and the structural design of the interdigital transducers are discussed. This paper focuses on analyzing the research status of physical flexible SAW sensors such as temperature, humidity, and ultraviolet radiation, including the sensing mechanism, bending strain performance, device performance parameters, advantages and disadvantages, etc. It also looks forward to the development of future chemical flexible SAW sensors for gases, the optimization of the direction of the overall device design, and systematic research on acoustic sensing theory under strain. This will enable the manufacturing of multifunctional and diverse sensors that better meet human needs.

## 1. Introduction

### 1.1. Surface Acoustic Wave Sensing Technology

In the 1880s, British physicist Raleigh accidentally discovered a type of sound wave with energy propagating along the surface of the earth while studying seismic waves, which were called surface acoustic waves (SAWs) [1]. In 1885, Raleigh published a paper titled “On Waves Propagated along the Plane Surface of an Elastic Solid”, in which he theoretically explained that, except the familiar bulk acoustic and shear waves, there was another type of surface wave propagating along an infinitely thick elastic body surface. This paper later became the beginning of SAWs.

After this discovery, more and more researchers paid attention to SAW technology, but there were no achievements until 1965 when R M. White and F.M. Voltmov invented the interdigital transducer (IDT), by which SAWs were excited on the surface of piezoelectric media [2]. SAW technology then became mature and was gradually improved. Nowadays, research on SAW technology and devices has been involved in various areas, such as acoustics, mechanics, and electronics. SAW technology has been widely applied in many fields including radar, communication, navigation, broadcasting, and television [3,4,5,6,7]. It is because, compared to other types of sensors, SAW technology not only has the advantages of high accuracy and sensitivity, facilitating large-scale production, a small size, being lightweight, and having a low power consumption but also realizes passive wireless detection easily. Therefore, researchers all over the world have recognized the potential value of SAW sensors and have begun to conduct much more extensive research on them [8,9].

There are two categories of SAW sensors: chemical sensors and physical sensors. SAW chemical sensors are essentially mass-type sensors that utilize for qualitative analysis the reversible adsorption between the selective sensitive film coated on the delay line during the propagation of the sound waves and the measured object. Quantitative detection is carried out by utilizing changes in the surface density or mass of the film, which cause changes in the wave velocity, frequency, and wavelength of the SAWs during the detection process [10]. The gas sensor, which is used to detect harmful gases, is one of the most important chemical sensors. In 1978, Wohltjen Henry and Dessy Raymond [3,4,5,6] published their first article on SAW gas sensors; over the past forty years, studies for detecting toxic gases, such as SO_2_, H_2_S, NO_2_, NH_3_, methane, hydrogen, explosives, chemical agents, etc., have been reported. For the SAW physical sensors, by inverse piezoelectric or piezoelectric effects, the input electrical signal is converted into an acoustic signal with input/output transducers. The most commonly used SAW physical sensors are temperature sensors, pressure sensors, strain sensors, and ultraviolet sensors, which have been applied in industries such as tire pressure monitoring systems (TPMS), power systems, and petrochemicals [11].

Because most SAW devices are prepared on piezoelectric thin films on rigid substrates including piezoelectric materials, glass, and silicon wafers, they are prone to deformation because of environmental changes, which ultimately leads to sensor performance degradation [12]. In recent years, with the continuous development of flexible functional materials, how to organically combine flexible technology with SAW sensors and expand their application areas has become a new research hotspot.

### 1.2. Flexible Technology

#### 1.2.1. Flexible Substrate Materials

Flexible technology is an emerging technology that has only recently been developed. As early as the 1960s, people had explored the use of flexible materials in flexible devices, such as flexible substrates, films, etc. In the early 21st century, flexible technology gradually emerged as a new type of technology in public. In 2013, researchers from the University of Illinois in the United States obtained the first patent for stretchable batteries [13], which greatly promoted the development of flexible technology. Subsequently, people around the world began to explore the potential of flexible technology, and flexible sensing has made significant progress.

Although the advantages of high integration and miniaturization allow traditional sensors based on rigid materials such as metal ceramics to be used in most environments, their rigid appearance limits the application area, such as healthcare, wearable devices, interactive robots, intelligent packaging, etc. Flexible sensing technology provides the possibility to solve these problems. The concept of flexible materials used in flexible sensing technology is relative to rigid materials, which have properties such as softness, low modulus, and easy deformation. Common flexible materials consist of polyvinyl alcohol (PVA), polyester (PET), polyimide (PI), polyethylene glycol naphthalene (PEN), paper, textile materials, etc. [14,15,16]. Sensors made by such flexible and ductile materials can effectively measure dynamic or shape-changing objects, and this is difficult for traditional rigid sensors to achieve.

Since the beginning of the 21st century, there has been significant progress in the research of flexible sensors. Initially, pressure sensor arrays on plastic films evolved into flexible sensors covering a wide range of physical and chemical sensing methods, including temperature, strain, electrophysiology, ions, biomarkers, metabolites, gases, etc., and the substrates used were no longer limited to early plastic films. At present, substrates such as ultra-thin plastic films, porous polymer pads/nets, elastic materials, and hydrogels have also been extensively studied [17].

#### 1.2.2. Types of Flexible Sensors

The technology of flexible sensing can be divided into two categories: one is the organic field effect transistor (OFET) technology, and the other is the surface acoustic wave (SAW) technology.

OFET sensing is the process by which the detected object affects the electrical signal of the OFET by changing the injection and transmission characteristics of the charge carriers. It achieves a sensing function through quantitative signal conversion. The device structure consists of two parts: a chemical layer and a physical converter. The main objective of the chemical layer is to adsorb the sensing object. The organic semiconductor in the sensing layer is the operating core, and the organic semiconductor can directly react selectively with the object [18]. Qualitative and quantitative analysis of the changes in the transistor current–voltage characteristic parameters is used to detect nitrogen dioxide or sulfur dioxide, and good results have been achieved in the treatment of toxic and harmful gases such as phosphorus, but OFET technology also has certain limitations; for example, sensors based on OFET are easily affected by the infiltration of H_2_O molecules in the environment, the doping effect of O_2_, or temperature-induced crystal structure differences, which can lead to changes in chemical properties. Alternatively, field-effect mobility and transistor on/off current ratio can be significantly reduced due to environmental differences.

Another type is the SAW flexible sensor. Because it is lightweight and flexible and has bendable characteristics, the flexible SAW sensor is a flexible electronic device that is promising and worth studying. Previous reports have shown that depositing or sputtering a layer of piezoelectric thin film on flexible organic polymers is the main way to prepare flexible SAW devices. However, in the complex manufacturing process of this sensor, it has been found that there is a severe lattice mismatch between the flexible substrate and the piezoelectric thin film, and there is a significant difference in the thermal expansion coefficient between the two. Therefore, the types of piezoelectric thin films and flexible substrates suitable for this sensor manufacturing are limited [19]. The most commonly used piezoelectric materials for developing thin film SAW devices on polymer substrates are ZnO and AlN. Although researchers have developed all organic SAW devices using polyvinylidene fluoride (PVDF), research on this material is still ongoing. Relatively speaking, the current mature method is to prepare ZnO/AlN piezoelectric thin films on PI/PEN. A study found that adding a multi-metal composite layer between the thin film and the substrate can improve the sensing performance of the current polymer substrate [20].

## 2. Flexible SAW Sensors

**Figure 1:**
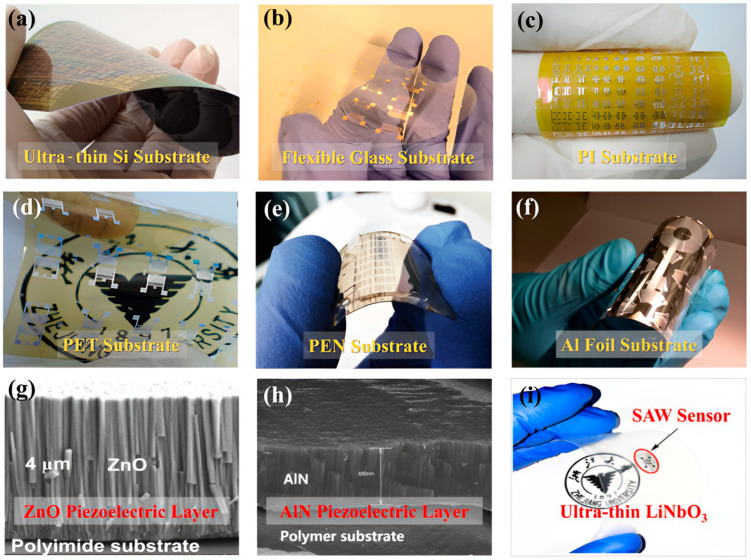
(**a**–**f**) Types of substrate materials used in flexible SAW devices [19]; (**g**,**h**) piezoelectric thin film used in flexible SAW devices [19]; and (**i**) ultra-thin LiNbO_3_ used in flexible SAW devices [19]. Copyright permission from AIP Publishing.

A flexible SAW device consists of three parts: a flexible substrate, electrodes, and piezoelectric materials. Flexible substrates are the foundation for developing SAW sensors. They are used to support crystal wafers and electrodes, and they serve as isolation and protection. In addition, in the different components of the SAW devices, the substrates control many important properties, including the mechanical stability and flexibility, electronic properties, thermal stability, surface smoothness, wettability, permeability, etc. Electrodes are generally composed of metals such as aluminum or gold, which are used to exert an electric field and to cause deformation of the crystal sheets. Piezoelectric materials are used to design the piezoelectric crystals or thin films, which are the core components of the SAW devices. By utilizing the piezoelectric effect of crystals, electrical signals are converted into SAWs, which propagate through the crystal surface and are ultimately converted into electrical signals for output. Currently, multiple research teams have validated the feasibility of this device design by improving these three components. Figure 1 shows the commonly used substrate materials and piezoelectric thin film materials for the preparation of flexible SAW sensors.

### 2.1. Design of SAW Devices Based on Different Flexible Substrates

Flexible SAW sensors can be broadly divided into two categories based on their manufacturing methods. One is SAW sensors that achieve their flexibility by reducing the thickness of the piezoelectric crystals. To reduce the thickness of the piezoelectric substrate, Dong Shurong’s team proposed using LiNbO_3_ thin films to manufacture flexible acoustic devices. Another approach is to combine piezoelectric thin films with flexible substrates to achieve flexibility in the device. To prepare piezoelectric thin film and flexible SAW devices, Luo (polymer flexible SAWs) [21], Fu (metal flexible SAWs) [21], and Zhou et al. (glass flexible SAWs) [22] have done much work.

#### 2.1.1. Flexible Thin Film SAWs on Polymer Substrates

Because traditional piezoelectric thin film SAW sensors use piezoelectric thin films to excite Rayleigh waves, the resonant frequency of the prepared devices is relatively low, which makes it difficult to adapt to high-frequency wireless passive environments. Therefore, the main substrate material for preparing flexible SAW sensors is organic polymers. In addition, sensors based on the Rayleigh wave mode have lower Q values due to the energy radiation caused by particle motion during application. Organic polymer substrates are different from traditional substrates. After etching the back of the flexible substrate deposited in the SAW working area, the flexible SAW device has high Q values and resonant frequencies and can be operated in high-frequency modes. There is not the phenomenon of radiation energy causing a decrease in the Q value like the SAW sensor in the Rayleigh wave mode during application. At present, many teams have prepared polymer-based flexible SAW sensors and conducted in-depth research on these substrate materials, as shown in Table 1. Table 1 lists the relevant physical and chemical properties of different polymer substrates, displaying their properties during preparation and the relevant experimental parameters after completion. It can be seen that polymer substrates such as PI, PET, and PEN have advantages over flexible glass, monocrystalline silicon, aluminum, and other metals.

**Figure 2:**
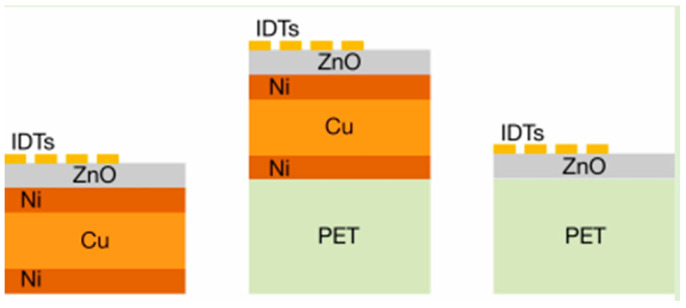
Substrate models with different metal and polymer compositions [20]. Reference [20] has been certified as open access by IEEE.

The application of polymers to SAWs first appeared in 2013. Jin et al. prepared flexible SAW temperature sensors by depositing piezoelectric ZnO thin films on the PI substrate. Since then, polymers have been discussed by the relevant scholars as the substrate choice for preparing flexible SAW sensors, including strain sensors, humidity sensors, gas sensors, UV sensors, PI, PET [27], and flexible glass. In 2013, He et al. prepared a ZnO/PI humidity sensor based on a flexible SAW and achieved a sensitivity of 3.5 KHz/% RH [28]. Good repeatability has been demonstrated under cycling conditions of humidity ranging from 5% to 87% rh without any surface treatment. In 2020, Tao et al. used a PI polymer as a transition layer to manufacture a flexible SAW biosensor based on polyimide-coated woven carbon fibers, and the wireless and passive characteristics and stability in continuous operation were demonstrated. However, its sensitivity to biological parameters under strain in complex environments is not ideal [29]. Therefore, when PI is used as a substrate, the factors affecting its sensitivity need to be considered. In 2014, He et al. used highly vertically arranged ZnO thin films on PET substrates to prepare SAW sensors that performed well even after 100 repeated bending cycles of up to 25,000 µε, proving that PET substrates still had good stability and repeatability when subjected to deformation [15]. In 2023, Shahrzad et al. studied a temperature sensor based on a ZnO thin film for the SAWs. The temperature sensor was coated with various substrates composed of metal thin layers (Ni/Cu/Ni) and/or polymers (polyethylene terephthalate, PET). They also compared and characterized the three different SAW devices manufactured, as shown in Figure 2. The research results showed that in examples of ZnO/PET the SAW devices prepared on polymer layers such as ZnO/Ni/Cu/Ni/PET exhibited enhanced temperature responsiveness, and the devices with larger wavelengths were more sensitive to UV exposure. In terms of driving purposes, the devices prepared on ZnO/Ni/Cu/Ni layers had the best acoustic current performance, while the devices prepared on ZnO/PET layers had no significant acoustic current effect. Through studying their acoustic flow performance on different substrates, it was found that adding a Ni/Cu/Ni metal layer between the ZnO and polymer layers might improve the sensing performance [20].

Polymer-based SAW sensors have broad potential for various applications, but there are still some development challenges that need to be addressed. One of the main challenges is to deposit high crystalline and piezoelectric layers on the substrate, which is crucial for achieving high device sensitivity and reliability. In addition, developing new organic polymer materials is another crucial factor to improve the performance of the SAW sensors. These new materials need to have better compatibility and shared mechanical properties with piezoelectric materials to improve the durability and sensitivity of the devices.

#### 2.1.2. Flexible Thin Film SAWs on Metal Substrates

Aluminum foil-based flexible SAW sensors have become another choice for manufacturing flexible SAW sensors due to their high thermal resistance and ease of processing [30]. However, aluminum metal foil has certain shortcomings, such as a high cost and a low impedance and opacity compared to polymers, and it may not be able to prepare stretchable devices or transmit high-frequency L-SAWs. Currently, researchers have begun to explore different technical approaches to overcome their limitations. In 2020, Tao et al. studied the performance of SAW sensors based on spin coated ZnO piezoelectric thin films on aluminum foil. By using a ZnO microstructure and nanostructure networks as sensing layers, the sensitivity of the flexible SAW sensors to ultraviolet (UV) radiation was improved. The sensitivity increased from −3.03 × 10^−6^ to −5.25 × 10^−6^ cm^2^ mW^−1^. Similarly, at 90% relative humidity, the humidity sensitivity increased by 2.9 times [31], indicating that metallic aluminum can indeed serve as a substrate for SAW devices.

The current Al-based flexible SAW devices typically have a large frequency temperature coefficient (TCF) of up to −760 ppm/K, which can be used for highly sensitive temperature sensing applications. However, Al foils have a high surface roughness, typically ~100 nm RMS, which is much larger than the standard thin glass (typically less than 1 nm RMS). This makes it difficult to manufacture high-frequency devices on metal foil. In addition, to ensure the electrical insulation and acoustic characteristics of the thin film SAW devices manufactured on these metal foils, they usually require polishing or coating with a thin film layer, which is a complex process. Therefore, metal-based flexible SAW devices are not the mainstream choice.

#### 2.1.3. Flexible Thin Film SAWs on Glass Substrates

**Figure 3:**
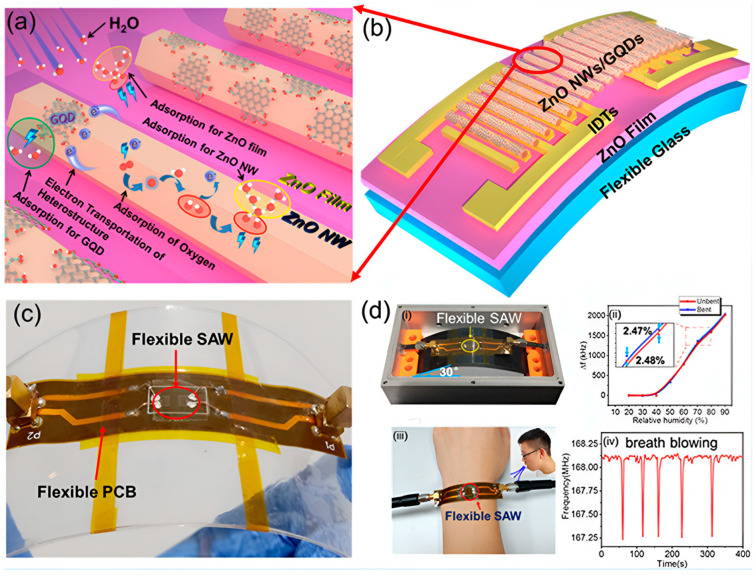
(**a**) Schematic diagram of the adsorption mechanism of H_2_O molecules on the composite sensing layer [22]. (**b**) Schematic diagram of the manufacturing process of SAW sensors based on ZnO, NW, and GQD [22]. (**c**) The device is packaged on a polyimide flexible printed circuit board (PCB) board and installed on a 1 mm thick polyethylene terephthalate (PET) [22]. (**d**) Schematic diagram of a testing system for humidity sensing [22]. (**i**) Photograph of the sensor at a bent state. (**ii**) Frequency shifts of a flexible humidity sensor (λ = 12 µm) under unbent and bent conditions at an angle of 30°. (**iii**) Illustration of the experimental setup of the flexible SAW for breathing detection on wrist. (**iv**) Resonant frequency changes in the flexible SAW humidity sensor after five cycles of exposure to breathing with a SAW device wavelength of 16 µm. Copyright permission from American Chemical Society.

Flexible glass is a term used for glass substrates with a thickness of less than 200 μm. Within this thickness range, glass exhibits good bendability and flexibility similar to plastic or metal foil substrates. Flexible glass retains all the advantages of block glass, such as high optical transmittance (over 90% under visible light), low stress, a smooth surface, good temperature resistance (up to 600 °C), a root mean square (RMS) roughness of 1 nm or less, a low coefficient of thermal expansion (CTE, ~4 × 10^−6^/°C), high dimensional stability, good chemical resistance, scratch resistance, and electrical insulation; as a result, it has good antioxidant and water resistance abilities. These excellent physical and chemical properties make flexible glass a viable choice for sensor substrates. Chen et al. prepared flexible/transparent ZnO thin film SAW devices on ultra-thin flexible glass substrates, and indium tin oxide (ITO) electrodes were also used on ZnO/glass [32] to form completely transparent and flexible devices. These glass-based flexible acoustic devices exhibited a similar performance to rigid glass devices but better light transmittance. In 2020, Professor Duan et al. from Hunan University developed a SAW humidity sensor based on a flexible glass substrate ZnO film. The developed sensor showed an ultra-high humidity sensitivity of 40.16 kHz/% RH and had good stability and repeatability. Figure 3 shows the adsorption mechanism of the humidity sensor and the design process of the device. The sensing system was constructed, and this flexible and transparent SAW sensor was further demonstrated to be of use for wearable electronic applications by using ultra-thin glass-based devices made entirely of inorganic materials. Humidity sensing and the detection of human respiration on a curved surface with a bending angle of 30° was possible. However, the stability cannot be guaranteed under acidic, alkaline, and high-temperature conditions [22].

Therefore, the team changed to depositing high-quality AlN film on a flexible substrate in 2022. A theoretical model was established using the coupled wave equation and boundary condition method to study the frequency response of its complex universal off-axis bending strain. The experiment results were consistent with the simulation results, and the effect was better than that of depositing ZnO thin films. This experiment was better proof of the universality and stability of the flexible glass substrate when facing different piezoelectric materials. By utilizing these flexible glasses, SAW devices have achieved a high sensitivity strain and humidity sensing. Compared with other SAW devices manufactured on flexible substrates such as polymer materials, SAW devices based on flexible glass have good mechanical and chemical properties, as well as low wave propagation losses. Some key properties of flexible glass are shown in Table 1. However, flexible glass is also very fragile, making it difficult to be listed as the main choice for flexible substrates.

#### 2.1.4. Flexible LiNbO_3_ Substrates

Most of the abovementioned substrates do not have piezoelectricity. Therefore, piezoelectric thin films are usually needed to enable acoustic devices. PVDF and LiNbO_3_ themselves are piezoelectric materials that can be used as substrates for piezoelectric thin films, or directly used to prepare flexible SAW sensors by reducing their thickness.

LiNbO_3_ has the advantages of a high electromechanical coupling coefficient and a relatively low cost due to mass production. However, as a new type of piezoelectric thin film material, there are few reports of its SAW sensor. In 2018, Xu et al. [33] developed a flexible SAW sensor on a single crystal 128°Y-cut LiNbO_3_ film using a bulk grinding/polishing technology, but their related performance was not systematically tested. Due to the current processing technology not meeting the requirements for stable bonding between LiNbO_3_ and SiO_2_ wafers, the film thickness could not reach 300 nanometers, so the SAW sensors made of LiNbO_3_ have a strong brittleness and are prone to breakage [34].

#### 2.1.5. Flexible PVDF Substrates

In addition to polymers and metal substrates, researchers are also constantly developing new substrate materials, such as PVDF. PVDF is a semi-crystalline polymer that exhibits four different phases, but only one phase, namely β, exhibits spontaneous polarization and piezoelectricity, which might be used to explore the feasibility of PVDF as a substrate for flexible SAW devices [35]. Reports have indicated that PVDF has good flexibility, low acoustic impedance, and can match the human body with a high piezoelectric constant [36]. Therefore, PVDF-based piezoelectric thin film sensors have been used for sleep monitoring. Due to its low cost, ease of processing, and excellent mechanical and electrical performance, PVDF is highly expected to become a disruptive solution for the development of fully organic and flexible SAW devices.

**Figure 4:**
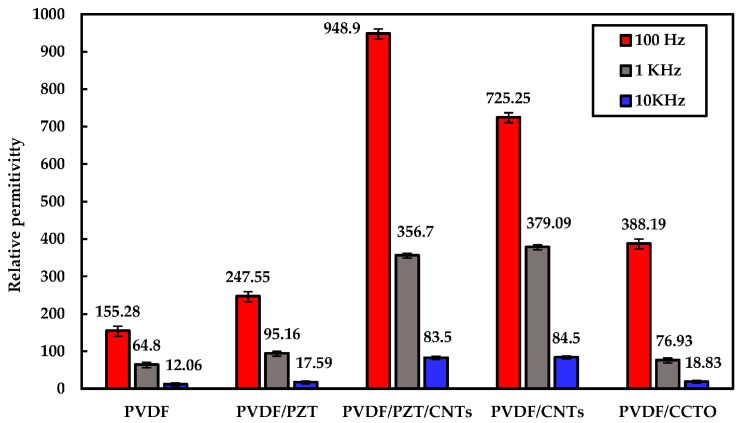
Frequency-dependent dielectric constant of PVDF mixed with different fillers [39]. Reference [39] has been certified as open access by MDPI.

**Figure 5:**
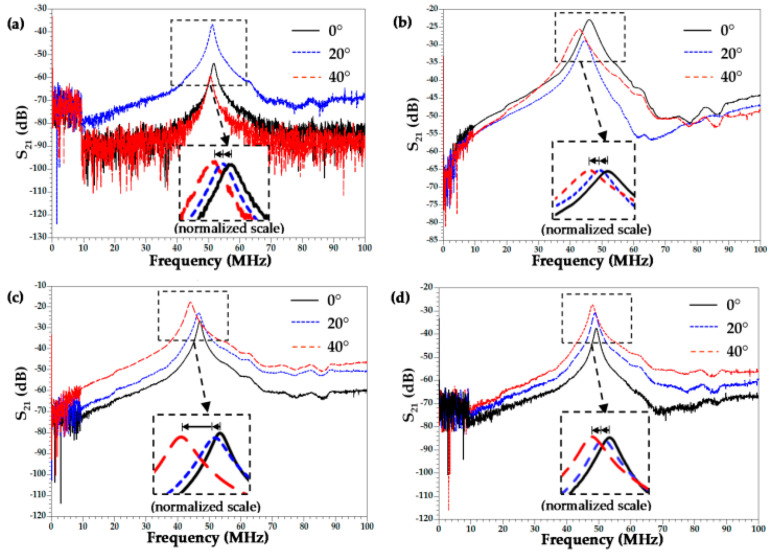
Frequency response representation from 10 kHz to 100 MHz at different angles: (**a**) PVDF/PZT [39]; (**b**) PVDF/PZT/CNTs [39]; (**c**) PVDF/CNTs [39]; and (**d**) PVDF/CCTO. The zoomed-in box shows the peak shift in the normalized frequency response when the SAW sensor was under bending to three different angles [39]. Reference [39] has been certified as open access by MDPI.

In 1997, the literature on Lamb wave PVDF transducers was first published [37]. In 2007, Preethichandra et al. studied the performance of PVDF in bending sensors [38]. When the PVDF substrate was bent, the PVDF sensor responded well to the bending curvature, and its amplitude and phase angle varied with the change in the bending curvature. In 2021, Rishikesh et al. [39] introduced a flexible SAW device prepared from PVDF, PZT micro materials, and CNT nanomaterials. The PVDF/CNTs composite yielded the maximum β fraction of 76.9%, as well as the maximum piezoelectric strain coefficient (d_33_) of 42 pC/N. Other composite types also yielded maximum property values: a 948.9 dielectric constant (ε_r_) by PVDF/PZT/CNTs and a 11.87 mVm/N piezoelectric voltage constant with PVDF/PZT. Mechanical strains were detected and measured by utilizing the change in wave characteristics, such as frequency shifts in the response from the fabricated SAW sensor. The research results showed a significant increase in the piezoelectric strain coefficient, voltage constant, and dielectric constant. Using the changes in the wave characteristics, such as the frequency offset in the response from the manufactured SAW sensor, mechanical strain could be detected. The results show that at a 40° bending angle the correlation between the measured frequency response and the quantitatively measured mechanical strain is as high as 15,800 µε. Figure 4 and Figure 5 show the changes in the relevant parameters and the comparison curves of SAW devices, which are relatively high for this specific application. Future work will focus on optimizing the IDT design to achieve better impedance matching and studying the basic peak response of the SAWs to object detection.

In 2022, K Prabakaran et al. [40] designed a highly sensitive SAW sensor modified with molecularly imprinted hydrophilic PVDF and selectively tested amino acids. The study found that within the concentration range of 0.5–150 ng/mL of L-tryptophan receptor, the sensor showed a linear response to concentration changes. The detection and quantification limits were calculated to be 0.2 ng/mL and 0.6 ng/mL, respectively. Moreover, MIP-SAW sensors with hydrophilic PVDF coatings exhibited an imprinting factor up to 9.34 on L-tryptophan.

Compared to other materials, PVDF also has some shortcomings, such as high manufacturing costs, difficult processing, and high prices. Although PVDF has good chemical corrosion resistance and UV resistance, it is also a brittle material that is prone to crack. In high-temperature or high humidity areas, changes in its physical properties can be caused, which might lead to performance damage. These shortcomings are the problems of PVDF that need to be solved as soon as possible.

#### 2.1.6. Other Flexible SAW Sensors

Monocrystalline silicon can also be used to make flexible SAW sensors. To achieve the flexible performance of SAW devices, the research on preparation schemes mainly focuses on reducing the thickness of the monocrystalline silicon. Zhang et al. proposed an application strategy for flexible acoustic devices based on a flexible AlN/Si structure (on 50 µm thick silicon), which has a large curvature bending radius (8 mm) [41]. However, the process of thinning silicon is relatively difficult and expensive, and, due to the brittleness of the silicon substrate, the device would be very fragile. Table 1 shows some key characteristics of flexible thin silicon.

In addition, there have been recent research reports on the piezoelectric properties of biopolymers such as cellulose and chitosan [42,43]. These materials have unique properties, including biocompatibility, renewability, and biodegradability, and have become an important, new research field for flexible SAW devices. Although the piezoelectric performance of such biopolymers is lower than that of synthesized organic polymers, they have shown potential in certain application fields.

### 2.2. Design of Flexible SAW Devices Based on Different Piezoelectric Materials

The materials used for piezoelectric thin films mainly include PZT, PVDF, ZnO, AIN, etc. [44,45], among which the materials used for preparing flexible surface acoustic wave devices are mainly ZnO and AlN. The materials used for flexible SAW sensors, including flexible substrates and piezoelectric thin films, are summarized in Table 2, and the device parameters of the physical sensors, such as the strain, ultraviolet radiation, temperature, and humidity, and the advantages, disadvantages, and manufacturing significance of each type of sensor are displayed.

From Table 2, it can be seen that piezoelectric thin films on the surface of flexible substrates is currently the mainstream choice for producing flexible SAW sensors, compared to other piezoelectric thin film materials such as ZnO and AlN, which have a wider range of applications. This is due to their advantages including a mature preparation process, stable physical and chemical properties, and their adaptability to the environment. Nowadays, the flexible SAW sensors produced are mainly physical sensors, which are used to measure parameters such as ultraviolet radiation, strain, humidity, etc. There is also some research and development on chemical sensors, such as for pH detection. However, the application of chemical flexible SAW sensors for gas detection is still in its early stages.

#### 2.2.1. ZnO-Based Flexible SAW Sensors

ZnO thin film is a kind of material with a high electromechanical coupling coefficient, low insertion loss, and good temperature stability. Its properties vary greatly with changes in doping components and preparation conditions. These differences make current research on ZnO thin films mainly focus on piezoelectric, gas-sensitive, and pressure-sensitive properties [52,53]. At present, ZnO thin films are mainly used for the development of SAW filters, while there were relatively few reports on their use in the design of flexible SAW devices. ZnO thin films were initially prepared by magnetron sputtering [27], and ZnO has been used as a piezoelectric thin film to prepare SAW sensors.

In 2013, Liu et al. [54] explored the use of ZnO/Al foil SAW devices for flexible acoustic fluids and reported similar studies on flexible SAW devices based on ZnO/Al foil. This was the first experiment to validate ZnO as a piezoelectric thin film for SAW sensors, which represented groundbreaking progress.

In 2014, He et al. [46] prepared a dual-mode (i.e., Rayleigh and Lamb modes) flexible ZnO/PI SAW UV sensor and studied its UV sensing performance. The UV sensitivities of these two modes were 111.3 and 55.8 ppm (mW cm^−2^)^−1^. In 2020, Wu et al. [22] developed a flexible transparent SAW humidity sensor based on ultra-thin glass using graphene quantum dot-modified ZnO nanowires as sensitive films. The sensor was able to detect human respiratory humidity in experiments, and the sensitivity of the device reached 40.16 KHz/% RH. The flexible SAW sensor was attached to a curved surface; the bending angle of the device was 30°; and its performance did not show any significant decrease.

**Figure 6:**
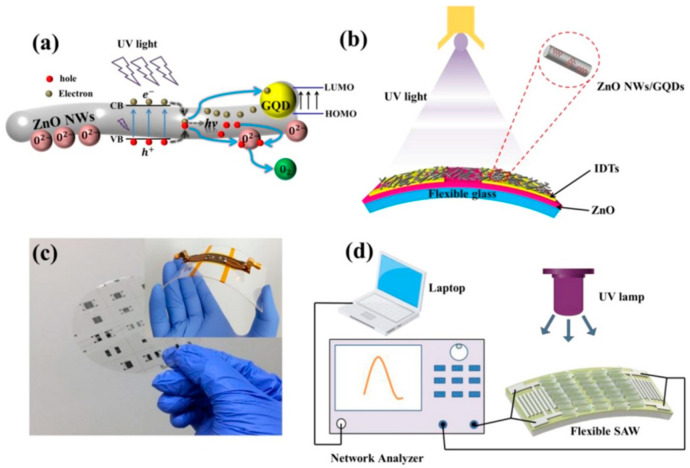
(**a**) Schematic diagram of the adsorption mechanism of ultraviolet radiation on ZnO composite films [55]. (**b**) Schematic diagram of device design for high flexibility transparent SAW ultraviolet (UV) photoelectric sensor [55]. (**c**) Schematic diagram of device preparation [55]. (**d**) Diagram of building the testing system [55]. Copyright permission from Elsevier.

In 2021, Yin et al. [55] prepared a new piezoelectric thin film by doping graphene on ZnO. They deposited the piezoelectric thin film on flexible glass to prepare a highly flexible transparent SAW ultraviolet (UV) photoelectric sensor. Specific details can be found in Figure 6. They found that the sensitivity of the sensor was three times higher than that of the previously developed polymer-based sensor. In addition, the sensor maintained a good performance 200 times at a bending angle of −30° without significant degradation, which demonstrated the excellent flexibility and stability of the ultraviolet photodetectors.

**Figure 7:**
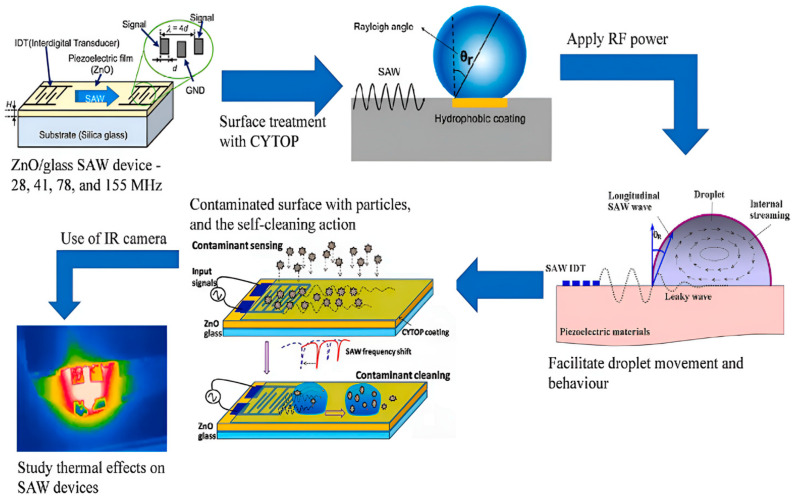
Working principle and specific structural diagram of SAW device surface cleaning [56]. Copyright permission from Elsevier.

In addition to doping aluminum and graphene, the structure of ZnO has also been improved. Recently, some people have used ZnO particle structures to prepare sensors instead of using thin film structures. In 2022, Huiling Ong et al. [56] used ZnO as a thin film to manufacture SAW sensors, studied the sound waves generated by the ZnO thin films on glass substrates, and explored the surface cleaning ability of transparent microfluidic devices using this sensor. Using ash particles and starch solution as model pollutants on the surface of the ZnO/glass SAW devices, they monitored the mass load of pollutants on the surface of the SAW devices with a high sensitivity of 280.0 ± 9.0 Hz/(μg/mm^2^). Based on the transmission of water droplets, the active surface cleaning of the pollutants was demonstrated, and the optimized SAW power was determined, which led to strong interactions between the water droplets and the pollutants, effectively cleaning the surface. The working principle and design concept of the device is available in Figure 7.

Subsequently, people were not satisfied with using only ZnO as a piezoelectric material, because its sensing performance and device performance were not ideal under certain conditions. Currently, people have started to incorporate other materials into the ZnO to improve the performance of the sensors. In 2023, Haekwan et al. [47] proposed a novel pH sensor with a center frequency of 230 MHz based on SAW sensors. To improve the sensitivity and stability of the sensor, the team incorporated ZnO nanoparticles into the sensitive layer. Compared with ZnO thin films, ZnO nanoparticles have the advantages of a low cost and a simple preparation process. As a result of different pH values, the change in the center frequency of the sensor could be observed. When it had a value of 1 μm, the frequency shift and linearity measured by the m-Thick ZnO NPs sensor between pH 7 and pH 2 were 144 kHz, and 0.957, respectively, indicating that the developed sensor exhibited a good chemical resistance and pH stability.

However, there are still many shortcomings in the preparation of thin films using ZnO for flexible SAW devices, such as the unstable chemical properties of ZnO, which are caused by strong chemical reactions when ZnO-based SAW sensors are exposed to high humidity conditions or operate underwater [21], which poses challenges to their stability and reliability. In addition, ZnO also exhibits significant dielectric loss and instability at high temperatures (above 500 °C).

#### 2.2.2. ALN-Based Flexible SAW Sensors

Aluminum nitride (AlN) has always been the mainstream piezoelectric thin film material due to its advantages of good mechanical properties, a low high-frequency loss, a good electric heating performance, better electrical insulation, an ideal piezoelectric performance, and a fast sound transmission speed [57]. In 1999, D. Manova et al. [58] used a DC magnetron reactive sputtering coating process to prepare a smooth, homogeneous, and crack-free 180 nm thick polycrystalline AIN film on low carbon steel and single crystal KCI substrates and continuously improved the process of ALN film. Compared with ZnO thin films, AlN-based SAW devices exhibit better chemical stability under acidic base conditions and are more suitable for harsh environments. In 2012, Jin et al. [59] studied the crystal structure of AlN thin films deposited on flexible PI by the reactive sputtering method and prepared corresponding flexible SAW devices. However, there are few reports on AlN-based SAW sensors.

In 2019, Leonardo et al. [60] studied the electro–acoustic optical response of SAW delay line devices sputtered with AlN thin films on Si and PEN substrates. This was the first report on the detection of ultraviolet radiation using a flexible AlN SAW device. In 2020, Leonardo et al. [48] prepared an AlN-based multimode SAW temperature sensor operating in the GHz range on a PEN substrate and compared the flexible device with the same SAW device manufactured on a silicon substrate. The results showed that the PEN-based SAW device had three working wave modes: Rayleigh, Love, and Lamb. Compared to the Rayleigh and Love waves, the Lamb wave mode exhibited a resonant frequency of up to 1.325 GHz, corresponding to a phase wave velocity of 10,600 m/s, 2.91% of electromechanical coupling, and a Q factor of 109. At the same time, the frequency temperature coefficient (TCF) values of the Rayleigh, Love, and Lamb waves were 149, 109, and 53 ppm/°C, respectively. The different behaviors of the three SAW modes provided the possibility of developing multiple sensing platforms in the same multi-mode device (such as temperature, microbial contamination, and light exposure). Thus far, there have been many studies using AlN for ultraviolet monitoring, but there is still a significant gap in practical application. In the same year, Lamanna et al. [16] prepared a SAW biosensor based on the AlN/PEN structure and applied it to the detection of escherichia coli based on protein a/antibody affinity. This Lamb wave device had the characteristics of high sensitivity and high phase wave propagation on polymer substrates and could estimate the mass of a single escherichia coli using the finite element method. In 2021, Leonardo et al. [61] studied the optical response of the device in the IR Vis UV range based on the above work. This was the first time that SAWs in the infrared visible ultraviolet range were used for optical response research, and a new mode of detecting ultraviolet light through flexible AlN-based SAW devices was proposed. In recent years, the structure of AlN has also been continuously improved. In 2021, Asseko et al. [62] proposed a two-dimensional finite element modeling and simulation method for SAW resonators based on Pt/AlN/Sapphire layered structures. Through characteristic parameters such as the Rayleigh wave phase velocity, electromechanical coupling coefficient, and quality factor, a finite element study was conducted on the relationship between the normalized thickness of AlN thin films and the performance of the resonators. Compared with the theoretical results in the literature, the results showed that the Rayleigh wave phase velocity of the device decreased from an initial value of 4360 m/s to the final value of 4085 m/s. Their work is not only helpful for the optimization of the design of future devices and the development of high-quality Q.K^2^ SAW resonators but also has the potential to improve the transmission distance of wireless SAW sensors. AlN-based SAW sensors have made progress not only in biological and physical aspects but also in chemical detection. At the same time, Piro et al. [49] developed a flexible AlN/PEN wearable PH SAW sensor with a center frequency of 500 MHz. They functionalized ZnO nanoparticles as pH-sensitive layers between IDTs and prepared SU-8 microfluidic channels. The sensor had a sensitivity of 30 KHz/PH in the range of PH 7 to PH 2.

There is not as much research on flexible sensing technology based on AlN piezoelectric thin films, because of the difficulty in controlling the deposition and texture of AlN thin films compared to ZnO thin films. For example, the oxygen or humidity in the deposition chamber and substrate orientation have a significant impact on the growth and microstructure of AlN thin films [63]. In addition, due to the high stress on the thin film, the requirement for ALN to form films with the thickness of a micrometer is higher; therefore, AlN thin films are more suitable for high-frequency operation [21], while ZnO thin films are more suitable for low-frequency operation devices, which is also one of the reasons for the lack of research reports on AlN.

#### 2.2.3. Other SAW Sensors

In addition to the abovementioned ZnO, AlN, and PVDF piezoelectric thin films, there are also some other thin film materials which are less commonly used, such as GaN, PZT, etc.

As the mainstream third-generation semiconductor material, GaN has attracted widespread attention in the industry due to its large bandgap, strong thermal conductivity, high-temperature resistance, and stable chemical properties. In 2018, F Bartoli et al. [64] studied the performance of AlN/IDT/GaN/Sapphire WLAW devices used as temperature sensors, which could be operated normally at 500 °C and have a temperature frequency coefficient (TCF) value of −34.6 ppm/°C. In 2023, Kim [65] reported a SAW sensor prepared from an independent single crystal piezoelectric GaN film that could be used as an electronic skin. They integrated an ultra-thin lightweight functional layer (gallium nitride and metal electrodes) into polydimethylsiloxane. These GaN SAW devices had good adhesion, long-term wear resistance, and a tensile strain that could reach 10.3%. They also demonstrated the wireless measurement of the carotid artery and pulse, which is a use of GaN thin films in the direction of strain sensors. Due to the high-temperature conditions required for the preparation of GaN films, it is impossible to directly prepare them on a flexible substrate, and only transfer fabrication methods can be used. However, the transfer process can easily lead to surface damage, resulting in an extremely low output power of the currently prepared devices. If these issues are not resolved, the application prospects of the GaN method are not optimistic.

PZT has the highest electromechanical coupling coefficient and piezoelectric constant among all piezoelectric thin film materials, but manufacturing a certain thickness of PZT thin film also requires optimization, which is currently difficult. Thus far, there have been few reports on using PZT thin films as SAW sensors. In 2021, Kiran Kumar Sappati et al. [66] printed acoustic sensors on thin and flexible PZT-PDMS (lead zirconate titanate polydimethylsiloxane) composite films using silver ink. The resonant frequency of the prototype resonator was 22.65 MHz, and the attenuation was −1.552 dBm. The mass sensitivity of the sensor measured using a PZT-PDMS layer reached −7.8 cm^2^/g, and the gas sensitivity to acetic acid and toluene concentrations was measured at 0.66 and 160.63 kHz/ppm, respectively. The detection limits for acetic acid and toluene were 10.9 and 0.03 ppm, respectively.

Due to the single property of the substrate material and the difficulty in refining the IDT finger width, SAW devices based on piezoelectric single crystals are unable to simultaneously meet the high-frequency, integration, and low-loss requirements of SAW devices. The composite thin film structure SAW device can compensate for the shortcomings of single crystal substrate SAW devices. By combining the different properties of thin film material layers, the mutual compensation of the performance can be achieved, and the thickness of each layer of thin film can be flexibly adjusted to optimize the performance of the SAW devices. In 2022, Wen et al. [67] designed an AlN/PZT composite piezoelectric thin film layer SAW device on a single crystal silicon substrate. The finite element method (FEM) was used to study the effect of the composite piezoelectric material thickness (the PZT thickness and the AlN thickness) on the phase velocity, electromechanical coupling coefficient, and electrode reflection coefficient of the zero-order and first-order SAWs in the AlN/IDT/PZT/Si structures. The optimal thin film thickness was obtained based on dispersion characteristics. The results indicated that by combining AlN thin films with PZT thin films and adjusting the thickness of the films, a combination of high sound velocity, high electromechanical coupling coefficient, and low electrode reflection coefficient could be achieved. This composite piezoelectric thin film structure could be applied in the design field of high-frequency and low-loss broadband SAW devices.

### 2.3. Flexible SAW Sensors Based on Different Electrode Structures

The electrode structure of SAW-based sensors typically consists of a pair of IDTs with opposite directions. One is served as the input IDT and the other is served as the output IDT. In the process of preparing flexible SAW sensors, the design of structures, such as forked electrodes, provides the possibility of improving the sensor performance. For flexible SAW sensors, the material and structure of the electrodes can affect the performance of the sensor.

Flexible electrode materials are a type of material with good mechanical flexibility and conductivity. Commonly used flexible electrode materials mainly include carbon materials, metal nanowires, and conductive polymers. Carbon materials include graphene, carbon nanotubes, and carbon fibers, and these materials have high conductivity and good flexibility, which can maintain conductivity in a bent state. Graphene has a unique two-dimensional structure, which can cause changes in the performance of sensors. Metal nanowires mainly include silver nanowires and copper nanowires with a high specific surface area. These materials can not only maintain good conductivity under a wide range of strains but the flexibility and conductivity can also be adjusted by controlling the morphology and density, thus meeting the needs of different application environments. Conductive polymers are polymer materials containing conductive functional groups, including polyaniline and polythiophene, which can be adjusted for conductivity through various methods, such as doping and synthesizing copolymers. Conductive polymers can maintain good conductivity in a bent state and have good solubility and processability, adapting to various shapes and sizes. At present, the flexible and stretchable properties of PI/PET, two flexible interdigital electrodes based on PI and PET, have been widely used in some fields such as human health detection.

**Figure 8:**
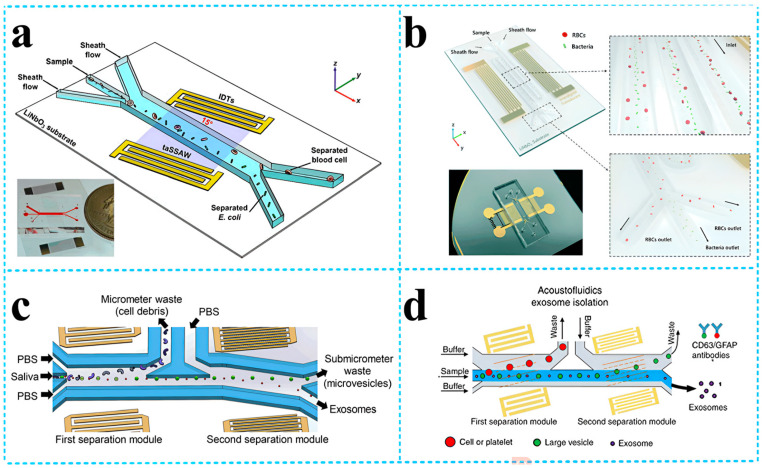
SSAW devices for the separation of other bioparticles. (**a**) Schematic of the acoustofluidic separation of *E. coli* from human blood samples using the taSSAW technique [68]; (**b**) the SSAW-enabled serpentine microfluidic device for high-throughput bacterial separation from human blood cells [68]; (**c**) schematic of the acoustofluidic device for salivary exosome separation [68]; and (**d**) schematic detailing the process of the isolation of exosomes from plasma samples [68]. Reference [68] has been certified as open access by the Royal Society of Chemistry.

**Figure 9:**
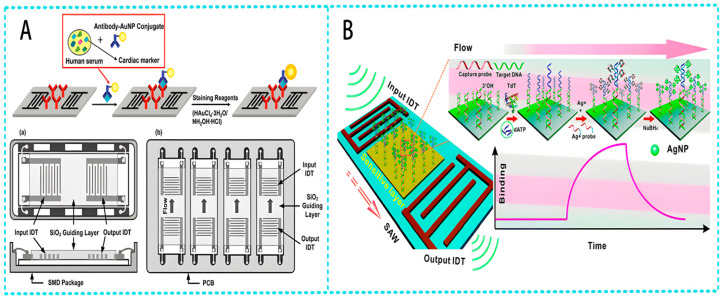
LW-SAW devices for biomolecule detection. (**A**) Schematic of the sandwich immunoassay format and LW-SAW sensor array for cardiac markers detection [68]. (**a**) Top and cross-sectional views of the single-packaged Love wave SAW sensor. (**b**) Top view of the Love wave SAW sensor array; and (**B**) schematic illustration of an SAW biosensor for highly specific and signal-amplified DNA detection [68]. Reference [68] has been certified as open access by the Royal Society of Chemistry.

In 2023, Wei et al. [68] classified and summarized SAW devices based on different vibration modes and boundary conditions. The team pointed out that SAW devices with guided wave layers that existed in IDT regions based on Rayleigh waves could limit acoustic energy to piezoelectric surfaces and had high sensitivity to surface modifications. They were the most commonly used liquid biosensing application platform and had advantages such as a fast reaction speed, small volume, and high sensitivity compared to traditional biological detection methods. Figure 8 shows the application of Rayleigh wave-based SAW separation technology for the separation of biological particles in a sample pretreatment. Flexible Lamb wave devices based on thin film structures have also been developed for liquid microfluidics and biosensing. Due to their advantages of a low cost, recyclability, and wireless monitoring, they have great potential application in wearable devices. Figure 9 shows the principle, device structure, and other details of the liquid biosensing platform based on Love waves applied in the biomolecular detection.

**Figure 10:**
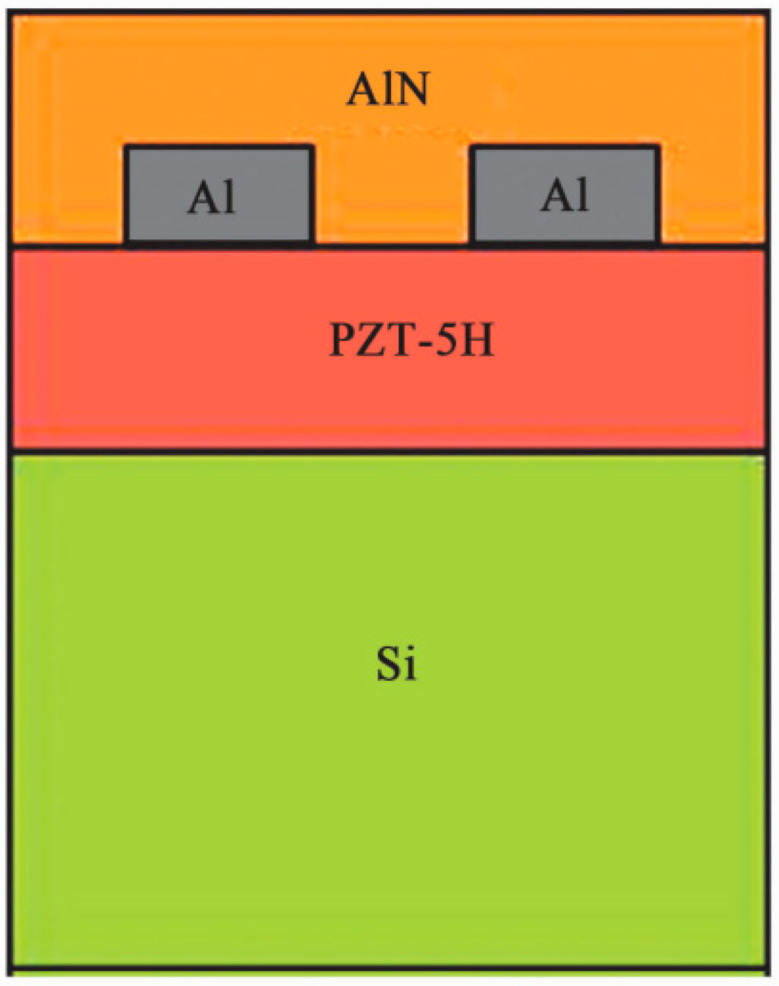
IDT periodic unit model structure [67]. Reference [67] has been certified as open access.

Thus far, most SAW devices are designed with IDT placed at the top. In 2015, Han et al. [69] found that placing IDT in a piezoelectric thin film layer resulted in a K^2^ of two to three times that of the traditional placement on the top surface in the ScAlN/diamond structure. The maximum K^2^ of Rayleigh waves could reach 14.5%, while that of the Xishawa waves could reach 10.0%. The IDT benefits from Al with good thermal conductivity, low density, and low acoustic impedance as the electrode material. The structure of the IDT periodic unit model is shown in Figure 10.

**Figure 11:**
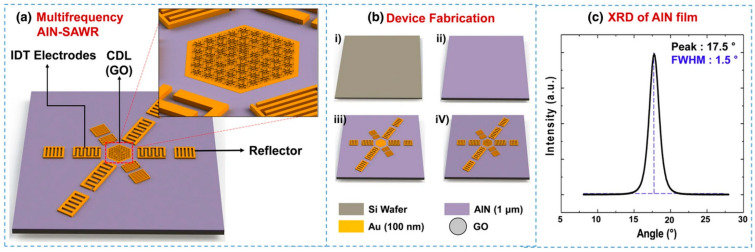
(**a**) The configuration of the SAWR consisting of IDTs, reflectors, and CDL [70]; (**b**) the fabrication process of the SAWR with a GO layer [70]. (**i**) a high-resistivity 6-inch silicon wafer. (**ii**) a 1-µm-thick AlN layer was sputtered on a high-resistivity 6-inch silicon wafer. (**iii**) Patterned 100 nm thick Au electrode on AlN layer. (**iv**) Selective modification of GO membrane on CDL.; and (**c**) an XRD analysis of the AlN layer [70]. Reference [70] has been certified as open access.

In 2019, researchers from the Gyeongbei Institute of Science and Technology (DGIST) in Daegu [70], South Korea proposed a multi-frequency AlN-SAWR single-chip integration process, which developed devices consisting of three pairs of interdigital transducers (IDTs), reflectors, and a single metalized electrode on a common delay line (CDL). Placing the CDL in the center between the three pairs of IDTs helped to form standing waves and mass sensitivity, and, then, the active/sensing area of the SAWR sensor formed. This single-chip multi-frequency SAWR system could be used to systematically analyze the correlation between the operating frequency and sensor performance. Researchers used this system to analyze the impact of graphene oxide content on sensor performance, accurately measuring the humidity field on fingers and saliva droplets produced during respiration. This provided a highly promising sensor system for human health monitoring and hygiene applications. Figure 11 illustrates the device structure and manufacturing process of the team’s AlN SAWR single-chip integrated process.

**Figure 12:**
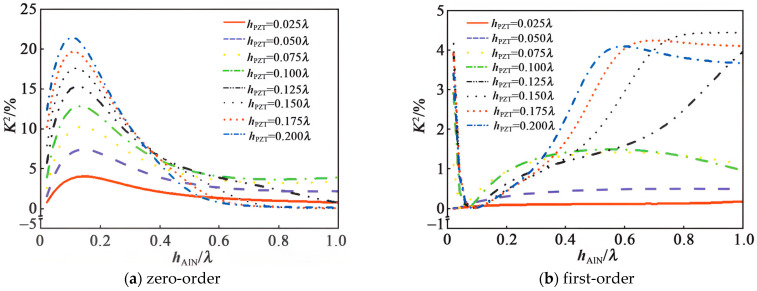
K^2^ dispersion characteristics of AlN/IDT/PZT/Si structure [67]. Reference [67] has been certified as open access.

In 2022, Wen et al. [67] designed an AIN/PZT composite piezoelectric thin film layer SAW device on a single crystal silicon substrate and achieved high K^2^ devices by placing the IDT in the piezoelectric thin film. From Figure 12, the K^2^ dispersion characteristic curve of the AlN/IDT/PZT/Si structure can be obtained, although it was difficult to meet the requirement of high electromechanical coupling coefficients of both the zero-order and first-order SAWs simultaneously. By adjusting the film thickness of the AIN and PZT at h_PZT_ = 0.2λ and h_ALN_ = 0.46λ, each K^2^ is greater than 3%.

## 3. Summary and Future Development

After more than 20 years of development, flexible sensing technology has made significant progress from stretchable batteries to today’s flexible sensors. Among them, flexible SAW sensors have more obvious advantages and potential application in terms of cost, accuracy, sensitivity, loss, or maturity compared to other types of flexible sensors. At present, flexible SAW devices have made tremendous progress in structural design, the selection and development of piezoelectric thin films, the selection of cross-finger electrode materials, and in structural design. More mature physical sensors have been developed, such as pressure, temperature, strain sensors, etc. However, research on chemical sensors is still in its early stages, and the detection objects that are detected are limited to humidity detection. Research on toxic gas detection is still empty, which is because, compared to rigid sensors, flexible sensors have varying acoustic propagation characteristics when subjected to deformation, and there are still many shortcomings in the development of high-performance flexible substrates. Today, flexible SAW sensing technology is facing some technical difficulties, which are troubling the relevant production solutions of most flexible sensors, such as flexible SAW gas sensors, and also the future development direction of flexible SAW technology.

(a) People are committed to designing a lightweight, stretchable, and compatible flexible substrate, but due to the easy loss of acoustic energy in flexible substrates, manufacturing high-performance (with reference to the electromechanical coupling coefficient and quality factor) surface acoustic wave devices on such flexible substrates is a very meaningful research direction. Based on the current development, developing new high-performance piezoelectric thin film materials (such as doped AlN or ZnO) with a high piezoelectric coefficient, high crystal orientation, low internal stress, and strong adhesion on flexible substrates is the mainstream method, because improving the quality of piezoelectric thin films is crucial for improving the Q factor and enhancing the performance of flexible SAW devices.

(b) In the process of making flexible sensors, it has been found that it is very difficult to make a thin film with a high piezoelectric coefficient on a flexible substrate. This is due to the lattice matching, high stress, and poor adhesion between the film and substrate. Therefore, the development of thin films and substrate materials and the analysis of their related physical properties are also meaningful. In addition, new piezoelectric thin film deposition methods should also be developed, because cutting and grinding techniques for the high-performance single crystal piezoelectric layers used in SAW devices are complex and expensive, and the utilization rate of larger materials in the manufacturing process is low, so the manufactured products not only cannot achieve complex deformation but are also prone to breakage.

(c) The sensing mechanisms of various flexible SAW sensors in the bending state are still being explored. The vibration mode of sound waves may change in the bending state, and multi-layer media will lead to the complexity of boundary conditions and the generation of more wave modes. The strain perturbation theory of different acoustic modes has not been systematically studied, and there is an urgent need for theoretical research and computational simulation. To achieve consistency in monitoring the results of planar and curved configurations, it is necessary to develop strain-insensitive flexible SAW devices, because the frequency shift of flexible SAW devices is significantly affected by strain and mechanical deformation. Proposing a method to extract key information from the scattering parameters of flexible SAW sensing, combining with machine learning or artificial intelligence (Al) methods to decouple the influence of multiple parameters, and then achieving the anti-strain interference effect of flexible SAW sensing on surface monitoring is a reliable method to realize the goal.

With the continuous research on flexible SAW sensor technology, the development of new flexible substrate materials and high-performance piezoelectric thin films, and the systematic study of device sound propagation characteristics under corresponding strain conditions, research content is becoming increasingly rich, which makes the produced flexible SAW sensors not only have the characteristics of miniaturization, integration, automation, etc. but also, in the future, have broad application prospects in the fields of informatization, unmanned technology in intelligence, and other fields, greatly promoting the development of flexible wearable sensors.

## Figures and Tables

**Table 1:** Relevant experimental parameters of different substrate materials [19]. Copyright permission from AIP Publishing.

Substrate	PI	PET	PEN	Flexible Glass	Thin Silicon	Al Foil
Glass transition temperature (T_g_)	155–360	70–110	120–155	/	/	/
Melting temperature (T_m_)	250–452	115–258	269	600	1410	660
Coefficient of thermal expansion (ppm c^−1^)	8–20	15–33	20	3.2	5	23.21
Water absorption rate (%)	1.3–3	0.4–0.6	0.3–0.4	/	/	/
Solvent resistance	good	good	good	good	good	fair
Transparency	poor	good	good	good	poor	poor
Dimensional stability	fair	good	good	good	good	good
Surface roughness (nm)	30	30	15	<0.5	<0.5	poor
References	[23]	[23,24]	[23,25]	[24,26]	[24,26]	[24]

**Table 2:** Experimental data of various flexible SAW sensors.

Device	Polymer Types	Piezoelectric Thin Film	Frequency (MHZ)	Sensitivity	Range of Detection or Strain Range	Advantages and Disadvantages	Ref.
Flexible SAW humidity sensor	PI	ZnO	Not mentioned	34.7 kHz/10% RH	5% RH to 87% RH	Good repeatability; the sensitivity increases with the decrease in wavelength; and improved characteristics of the sensors with shorter wavelengths.	[28]
Flexible SAW UV sensor	PI	ZnO	14.95 (R)	0.85 KHz/(mW/cm^2^)	0 mW/cm^2^ to 151.2 mW/cm^2^	The device can achieve multifunctional sensing functions in liquid environments.	[29]
Dual resonant mode flexible SAW sensor	PET	ALN	Not mentioned	Not mentioned	2500 με for 100 times	The SAW devices are highly sensitive to compressive and tensile strains, and they are particularly suitable for sensing large strains.	[15]
Flexible SAW humidity sensor	flexible glass	ZnO, ALN	290.1, 211.8, 166.9, 139	180.08, 130.29, 99.50, 64.38 Hz/με	0–1332 με	ZnO-based SAW devices are not stable in extremely acidic and alkali environments along with high-temperature environments. AlN/glass flexible-based SAW devices have never been reported.	[22]
Flexible SAW strain sensor	LiNbO_3_	LiNbO_3_	162–325	193 Hz/με	±3500 με	No visible deterioration was observed after cyclic bending for hundreds of times, showing desirable stability and reliability.	[33]
Flexible SAW devices using indium tin oxide (ITO) electrodes	flexible glass	ZnO	133.2	34.7 Hz/με	±3000 με	The strain sensors demonstrated excellent stability and reliability under cyclic bending. The flexible glass-based SAW devices have a better optical transmittance than those on rigid glass.	[46]
A new pH sensor based on SAWs	Not mentioned	ZnO	230	Not mentioned	PH7 to PH2	A low-cost, simple structure and good mechanical properties. However, they still suffer from a low sensitivity, low chemical resistance, and poor stability due to the piezoelectric.	[47]
The flexible SAW sensor	PEN	ALN	190 (R)500 (L)	577 Hz/με (R)1156 Hz/με (L)	429 (R)181 (L)	This polymeric-based flexible SAW device could pave the way for the development of a passive wireless strain sensor.	[48]
A novel aluminum nitride conformable SAW immunosensor	PEN	ALN	Not mentioned	6.54 × 10^5^ CFU/mL	Not mentioned	This work demonstrates the high biosensing potential of flexible polymeric SAW devices for the control of bacteria contamination in the food chain, water, and smart packaging.	[16]
Flexible and biocompatible pH sensor based on SAWs	PEN	ALN	500	30 kHz/pH	PH7 to PH2	This is the first time a microfluidic channel has been directly fabricated on a SAW flexible device for sensing applications.	[49]
Flexible SAW sensor	PEN	ZnO	110.7	130 Hz/με	2500 με	The SAW devices are also highly sensitive to compressive and tensile strains, exhibiting excellent anti-strain deterioration properties; thus, they are particularly suitable for sensing large strains.	[15]
SAW flexible strain sensor	Quartz AT-X	Not mentioned	156.16, 260.15	186.2, 251.9 Hz/με	5000 με	An excellent linearity; the new sensor paves the way for the fabrication of high-performance strain-sensing devices.	[50]
A SAW flexible strain sensor	PMN-PT	Not mentioned	129	1243 Hz/με	±1000 με	This developed sensor is an excellent candidate for small stain measurements, and it also can realize a wide strain measurement range from −1000 με to +1000 με.	[51]
